# Enhancement of the anti-tumour effect of cyclophosphamide by the bioreductive drugs AQ4N and tirapazamine

**DOI:** 10.1054/bjoc.1999.1132

**Published:** 2000-03-21

**Authors:** O P Friery, R Gallagher, M M Murray, C M Hughes, E S Galligan, I A McIntyre, L H Patterson, D G Hirst, S R McKeown

**Affiliations:** Radiation Science Research Group, School of Biomedical Sciences, University of Ulster at Jordanstown, Newtownabbey, Co. Antrim, BT37 0QB, Northern Ireland, UK; School of Pharmacy, University of London, Brunswick Square, London, WC1N 1AX, UK

**Keywords:** AQ4N, tirapazamine, cyclophosphamide, anti-tumour effect

## Abstract

The ability of the bioreductive drugs AQ4N and tirapazamine to enhance the anti-tumour effect of cyclophosphamide was assessed in three murine tumour models. In male BDF mice implanted with the T50/80 mammary carcinoma, AQ4N (50–150 mg kg^−1^) in combination with cyclophosphamide (100 mg kg^−1^) produced an effect equivalent to a single 200 mg kg^−1^dose of cyclophosphamide. Tirapazamine (25 mg kg^−1^) in combination with cyclophosphamide (100 mg kg^−1^) produced an effect equivalent to a single 150 mg kg^−1^dose of cyclophosphamide. In C3H mice implanted with the SCCVII or RIF-1 tumours, enhancement of tumour cell killing was found with both drugs in combination with cyclophosphamide (50–200 mg kg^−1^); AQ4N (50–200 mg kg^−1^) produced a more effective combination than tirapazamine (12.5–50 mg kg^−1^). Unlike tirapazamine, which showed a significant increase in toxicity to bone marrow cells, the combination of AQ4N (100 mg kg^−1^) 6 h prior to cyclophosphamide (100 mg kg^−1^) resulted in no additional toxicity towards bone marrow cells compared to that caused by cyclophosphamide alone. In conclusion, AQ4N gave a superior anti-tumour effect compared to tirapazamine when administered with a single dose of cyclophosphamide (100 mg kg^−1^). © 2000 Cancer Research Campaign


*British Journal of Cancer* (2000) **82**(8), 1469–1473
© 2000 Cancer Research Campaign
DOI: 10.1054/ bjoc.1999.1132, available online at http://www.idealibrary.com on IDEAL®

### Enhancement of the anti-tumour effect of cyclophosphamide by the bioreductive drugs AQ4N and tirapazamine

**OP Friery[1], R Gallagher[1], MM Murray[1], CM Hughes[1], ES Galligan[1], IA McIntyre[1], LH Patterson[2], DG Hirst[1] and SR McKeown[1]**

[1]Radiation Science Research Group, School of Biomedical Sciences, University of Ulster at Jordanstown, Newtownabbey, Co. Antrim, Northern Ireland, BT37 0QB, UK; [2]School of Pharmacy, University of London, Brunswick Square, London WC1N 1AX, UK

**Summary** The ability of the bioreductive drugs AQ4N and tirapazamine to enhance the anti-tumour effect of cyclophosphamide was assessed in three murine tumour models. In male BDF mice implanted with the T50/80 mammary carcinoma, AQ4N (50–150 mg kg$^{-1}$) in combination with cyclophosphamide (100 mg kg$^{-1}$) produced an effect equivalent to a single 200 mg kg$^{-1}$ dose of cyclophosphamide. Tirapazamine (25 mg kg$^{-1}$) in combination with cyclophosphamide (100 mg kg$^{-1}$) produced an effect equivalent to a single 150 mg kg$^{-1}$ dose of cyclophosphamide. In C3H mice implanted with the SCCVII or RIF-1 tumours, enhancement of tumour cell killing was found with both drugs in combination with cyclophosphamide (50–200 mg kg$^{-1}$); AQ4N (50–200 mg kg$^{-1}$) produced a more effective combination than tirapazamine (12.5–50 mg kg$^{-1}$). Unlike tirapazamine, which showed a significant increase in toxicity to bone marrow cells, the combination of AQ4N (100 mg kg$^{-1}$) 6 h prior to cyclophosphamide (100 mg kg$^{-1}$) resulted in no additional toxicity towards bone marrow cells compared to that caused by cyclophosphamide alone. In conclusion, AQ4N gave a superior anti-tumour effect compared to tirapazamine when administered with a single dose of cyclophosphamide (100 mg kg$^{-1}$). © 2000 Cancer Research Campaign

**Keywords**: AQ4N; tirapazamine; cyclophosphamide, anti-tumour effect

The complete eradication of solid tumours by chemotherapy agents is often compromised by the presence of treatment-resistant hypoxic cells within a tumour (Kennedy, 1987). Several bioreductive drugs have been developed to overcome this problem. These agents are prodrugs which undergo metabolic reduction in hypoxic cells and, therefore, specifically target this subpopulation (Workman and Stratford, 1993). Combination of bioreductive drugs with radiation has shown a beneficial interaction, e.g. tirapazamine (TPZ: 3-amino-1,2,4-benzotriazine-1,4-dioxide) and RB6145 (α[(2-bromoethyl)amino-2-hydroxypropyl}-2-nitroimidazole) have both been shown to enhance the anti-tumour effect of X-rays (Brown and Lemmon, 1990; Adams et al, 1992). More recently bioreductive drugs have been combined with cancer chemotherapy agents. Tirapazamine has been shown to potentiate the anti-tumour effect of a range of chemotherapy drugs including cyclophosphamide and cisplatin (Langmuir et al, 1994; Siemann, 1996; Dorie and Brown, 1997). RB6145 has been shown to enhance the anti-tumour effect of lomustine and cyclophosphamide (Siemann, 1994).

AQ4N (1,4-Bis{[dimethylamino-N-oxide)ethyl]amino}5,8-dihydroxy-anthracene-9,10-dione) is a novel bioreductive drug which is metabolized in hypoxic conditions to AQ4, a cytotoxic metabolite which binds with high affinity to DNA and is a potent inhibitor of DNA topoisomerase II (Patterson, 1993; Smith et al, 1996). Previous studies have shown that AQ4N can significantly enhance the anti-tumour effect of both single and fractionated doses of radiation (McKeown et al, 1995, 1996).

This paper aims to assess the ability of AQ4N to enhance the anti-tumour effect of cyclophosphamide in three murine tumour models and to compare this to the efficacy of tirapazamine as a potentiator of cyclophosphamide. In addition, the toxic effect of AQ4N and tirapazamine on normal bone marrow will be assessed using a spleen colony assay.

*Received 25 August 1999*
*Revised 1 December 1999*
*Accepted 3 December 1999*

*Correspondence to:* SR McKeown

### MATERIALS AND METHODS

### Tumour models

The T50/80 tumour is a poorly differentiated mammary carcinoma which arose in a female B6D2F1 mouse (Moore et al, 1988). Tumours were implanted intradermally (i.d.) on the rear dorsum of male BDF mice aged 8–12 weeks using early passages (4–12) of the tumour. Male C3H mice aged 8–12 weeks were implanted i.d. with SCCVII or RIF-1 tumour cells. Each mouse received $2 \times 10^5$ tumour cells in 0.05 ml phosphate-buffered saline (PBS). Tumour stocks of SCCVII and RIF-1 cells were maintained by the protocol of Twentyman et al (1980). All animal experiments were carried out in accordance with the UK Animals (Scientific Procedures) Act 1986.

### Drug preparation and administration

AQ4N (15 mg ml$^{-1}$) and cyclophosphamide (5 mg ml$^{-1}$) were made up in distilled water. Tirapazamine (3 mg ml$^{-1}$) was prepared

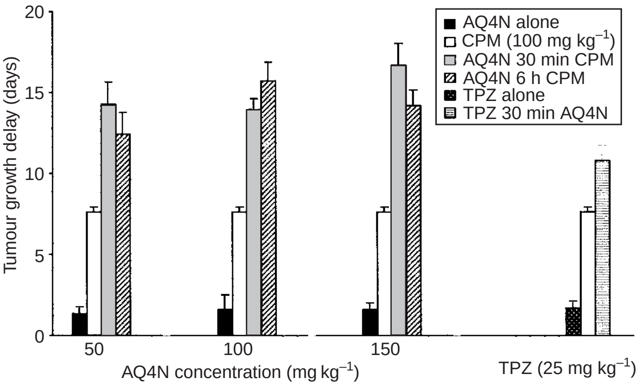

**Figure 1** Interaction of the bioreductive drugs AQ4N and tirapazamine (TPZ) with cyclophosphamide (CPM) in the T50/80 tumour model. AQ4N (50–150 mg kg$^{-1}$) was administered as a single i.p. injection 30 min or 6 h prior to cyclophosphamide (100 mg kg$^{-1}$). Tirapazamine (25 mg kg$^{-1}$) was administered as a single i.p. injection 30 min prior to cyclophosphamide (100 mg kg$^{-1}$). Results were obtained from six to nine tumours per treatment group. Values are means ± s.e.

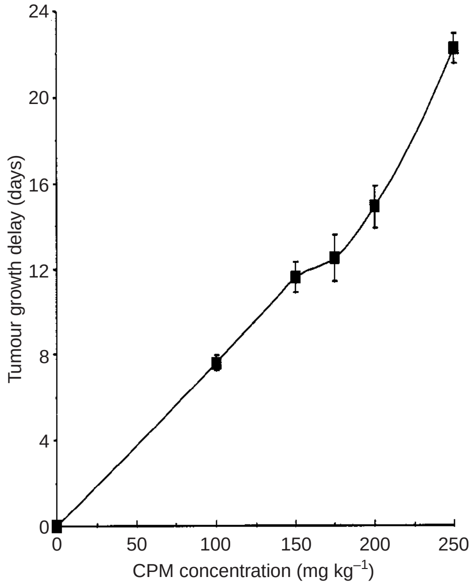

**Figure 2** Dose–response curve for cyclophosphamide (CPM). BDF mice bearing the T50/80 tumour were treated with a single i.p. dose of cyclophosphamide (100–250 mg kg$^{-1}$). Results were obtained from six to nine tumours per treatment group. Values represent mean ± s.e.

in PBS and due to its low solubility it was dissolved by sonicating for 20 min (McAleer et al, 1992). All drugs were administered as a single intraperitoneal (i.p.) injection. Systemic toxicity was monitored by measuring animal weight three times weekly. Cyclophosphamide was administered as a single i.p. injection (0–250 mg kg$^{-1}$) in order to obtain a dose–response curve. For combined treatments AQ4N (50–150 mg kg$^{-1}$) or tirapazamine (25 mg kg$^{-1}$ or 50 mg kg$^{-1}$) were administered 30 min prior to a single i.p. injection of cyclophosphamide (50–100 mg kg$^{-1}$). In some experiments this time interval was increased to 6 h or 24 h for AQ4N and 150 min or 24 h for tirapazamine (see Results for details).

### Measurement of tumour response to treatment: tumour growth delay

BDF mice were treated when the tumour reached 6.5–7.5 mm GMD (geometric mean of three orthogonal diameters). Tumours were measured three times weekly and the time taken to reach double its treatment volume (VDT) was used as a measure of anti-tumour efficacy. Tumour growth delay (TGD) was calculated by subtracting the mean VDT for control tumours from that obtained after drug exposure. The effect on tumour growth was assessed using the analysis of variance test (ANOVA).

### Measurement of tumour response to treatment: in vivo–in vitro clonogenic assay

C3H mice bearing SCCVII and RIF-1 tumours were treated with drug combinations when the tumours reached a volume of 100–200 mm$^3$. After 24 h the tumours were excised, minced and digested with an enzyme cocktail (6 mg DNAase, 2 mg collagenase and 2 mg pronase per 10 ml PBS) to produce a single cell suspension. The cells were counted, diluted as necessary, plated and incubated at 37°C in 95% air/5% carbon dioxide for 12–14 days. Colonies were fixed and stained with 0.4% crystal violet in methanol and then scored by eye. Surviving fraction (SF) was calculated as the number of colonies counted divided by the number of cells plated for a given treatment, divided by the same fraction determined for untreated control tumours.

### Normal tissue toxicity

The response of a normal tissue to AQ4N and tirapazamine in combination with cyclophosphamide was evaluated in the bone marrow of BDF mice by the spleen colony assay (Till and McCullough, 1961). Donor mice were injected i.p. with various doses and combinations of the drugs (see Results for details). Twenty-four hours later, bone marrow cells were isolated from the femurs of donor mice by flushing through PBS (0.7 ml × 2). Bone marrow cells from six mice were pooled, counted and injected into the tail vein of six recipient BDF mice, which had been whole body irradiated with 9 Gy 24h previously. The spleens were removed from the recipient mice 8 days later, and immersed in Bouin's stain for 1.5 h prior to fixation in formalin. Spleen colonies were counted by eye and surviving fraction was calculated as before. Statistical significance was determined by the Student's *t*-test.

### RESULTS

### The anti-tumour effect of drug combinations: T50/80 tumour model

AQ4N showed only a minimal inhibition of tumour growth at all doses (Figure 1). When AQ4N was administered 30 min prior to cyclophosphamide (100 mg kg$^{-1}$), T50/80 tumour growth delay ranged from 14.2 days (50 mg kg$^{-1}$ of AQ4N) to 16.7 days (150 mg kg$^{-1}$ of AQ4N), (Figure 1). A similar effect was observed when the time interval between administration of the two drugs was increased to 6 h. Statistical analysis showed that combining

© 2000 Cancer Research Campaign

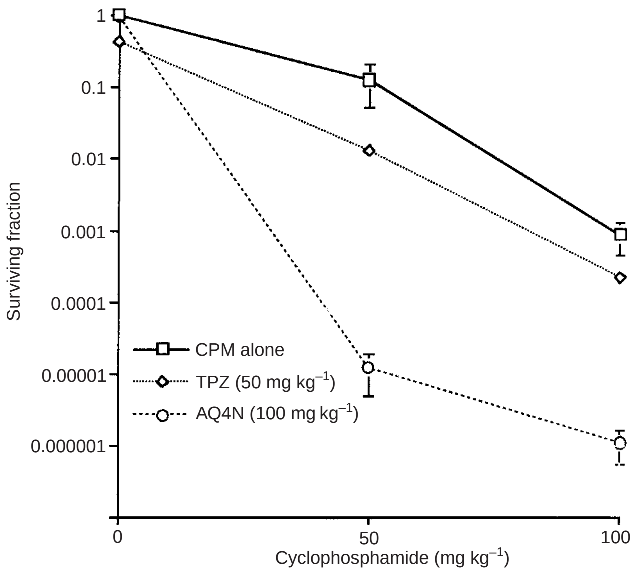

**Figure 3** Interaction of the bioreductive drugs AQ4N and tirapazamine (TPZ) with cyclophosphamide (CPM) in the SCCVII tumour model. AQ4N (100 mg kg$^{-1}$) and tirapazamine (50 mg kg$^{-1}$) were administered as a single i.p. injection 30 min prior to CPM (100 mg kg$^{-1}$). Tumours were excised 24 h after treatment and clonogenic cell survival measured. Six to nine tumours were treated in each group

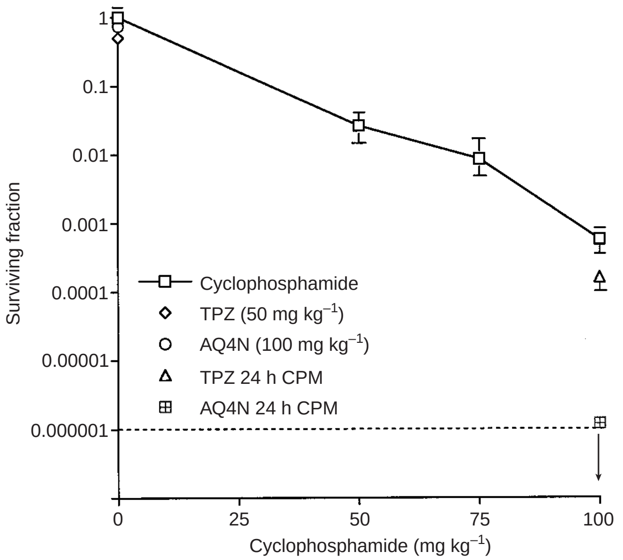

**Figure 4** Interaction of the bioreductive drugs AQ4N and tirapazamine (TPZ) with cyclophosphamide (CPM) in the RIF-1 tumour model. AQ4N (100 mg kg$^{-1}$) or TPZ (50 mg kg$^{-1}$) was administered as a single i.p. injection 30 min prior to CPM (100 mg kg$^{-1}$). Tumours were excised 24 h after treatment and clonogenic cell survival measured. Six to nine tumours were treated in each group

**Table 1** The anti-tumour effect of AQ4N administered at three different times prior to cyclophosphamide in the SCCVII and RIF-1 tumours

| | Surviving fraction (SF) | |
|---|---|---|
| **Treatment** | **Rif-1** | **SCCVII** |
| CPM (100 mg kg$^{-1}$) | $3.43 \times 10^{-4}$ | $8.80 \times 10^{-4}$ |
| AQ4N 30 min prior to CPM | $< 1.0 \times 10^{-6}$ | $< 1.0 \times 10^{-6}$ |
| AQ4N 6 h prior to CPM | $< 1.0 \times 10^{-6}$ | $< 1.0 \times 10^{-6}$ |
| AQ4N 24 h prior to CPM | $< 1.0 \times 10^{-6}$ | $< 1.0 \times 10^{-6}$ |

Each of the drugs was administered i.p. as a single dose. AQ4N (100 mg kg$^{-1}$) was administered 30 min, 6 h and 24 h prior to cyclophosphamide (100 mg kg$^{-1}$). The anti-tumour effect was assessed by the clonogenic assay and the surviving fraction calculated. Results were obtained from 6–9 tumours per treatment group.

AQ4N (50–150 mg kg$^{-1}$) with cyclophosphamide resulted in a significant increase in anti-tumour effect compared with cyclophosphamide alone ($P$ = 0.0001). There was no significant difference between results obtained using the 30 min or 6 h treatment intervals ($P$ = 0.15). In both cases, the anti-tumour effect of AQ4N (100 mg kg$^{-1}$) in combination with cyclophosphamide (100 mg kg$^{-1}$) was found to be approximately equivalent to a single 200 mg kg$^{-1}$ dose of cyclophosphamide (TGD = 14.9) (Figure 2).

Tirapazamine (25 mg kg$^{-1}$) alone had only a limited effect on tumour growth. When tirapazamine (25 mg kg$^{-1}$) was combined with cyclophosphamide (100 mg kg$^{-1}$), the TGD was calculated as 10.8 days (Figure 1). This was significantly better than the anti-tumour effect seen with cyclophosphamide alone ($P$ = 0.0013) and was approximately equivalent to the effect of a 150 mg kg$^{-1}$ dose of cyclophosphamide alone (Figure 2).

### The anti-tumour effect of drug combinations: SCCVII tumour model

The anti-tumour effect of AQ4N or tirapazamine in combination with cyclophosphamide was evaluated in the SCCVII tumour using an in vivo–in vitro clonogenic assay. Cyclophosphamide gave almost 3 logs of cell kill at the higher dose tested (100 mg kg$^{-1}$). Combination of tirapazamine (50 mg kg$^{-1}$) administered 30 min before cyclophosphamide at each dose caused an increase in tumour cell kill of about 1 log (Figure 3). AQ4N (100 mg kg$^{-1}$) administered either 30 min, 6 h or 24 h prior to cyclophosphamide at each dose resulted in an increased cell kill of at least 3 logs (Figure 3; Table 1). Changing the schedule with AQ4N showed no appreciable difference in the anti-tumour effect with the maximal cell kill obtained even if the interval between cyclophosphamide and AQ4N administration was 24 h (Table 1).

### The anti-tumour effect of drug combinations: RIF-1 tumour model

The effect of AQ4N in combination with cyclophosphamide was also investigated in the RIF-1 model. The results are similar to those observed in the SCCVII model, with AQ4N enhancing the anti-tumour effect of cyclophosphamide by greater than 3 logs of cell kill (Figure 4). This is an underestimation of the anti-tumour effect as no colonies were detected with $10^6$ cells. Again the time scheduling of AQ4N made no difference to the overall anti-tumour effect observed (Table 1). Tirapazamine (50 mg kg$^{-1}$) caused only a slight increase in anti-tumour effect when administered 24 h prior to cyclophosphamide (100 mg kg$^{-1}$) (Figure 4).

### Normal tissue toxicity: spleen colony assay

AQ4N was found to be less toxic than tirapazamine towards bone marrow cells. AQ4N alone, even at the highest dose, i.e.

© 2000 Cancer Research Campaign 

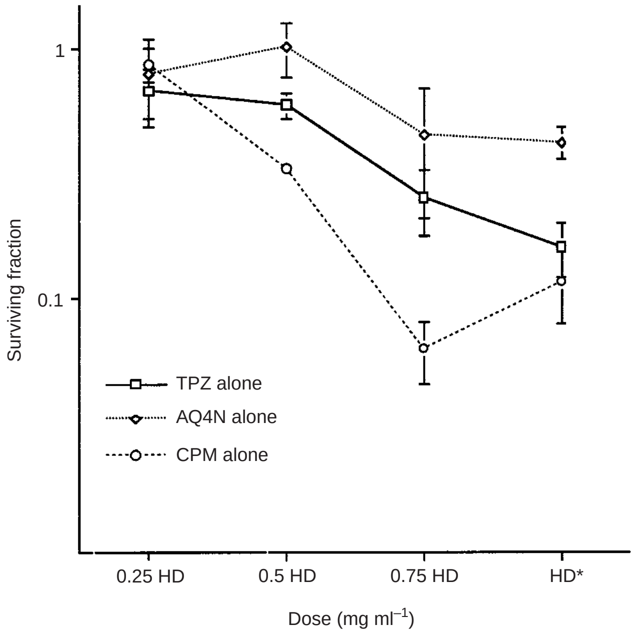

**Figure 5** The effect of AQ4N, tirapazamine (TPZ) and cyclophosphamide (CPM) on bone marrow toxicity. BDF mice were dosed i.p. with a range of doses: AQ4N (50–200 mg kg$^{-1}$), TPZ (12.5–50 mg kg$^{-1}$) and CPM (50–200 mg kg$^{-1}$). Survival of bone marrow cells was assessed by the spleen colony assay. To allow drug doses to be plotted on the same scale, they are plotted with the highest dose designated as HD. Results are the means ± s.e for six mice

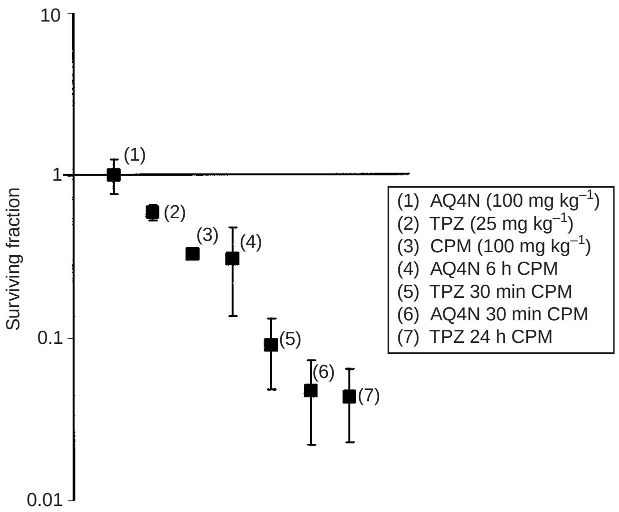

**Figure 6** The effect of AQ4N and tirapazamine (TPZ) on the bone marrow toxicity of cyclophosphamide (CPM). BDF mice were dosed i.p. with AQ4N (100 mg kg$^{-1}$) 30 min and 6 h prior to CPM (100 mg kg$^{-1}$). TPZ (25 mg kg$^{-1}$) was administered 30 min and 24 h prior to CPM (100 mg kg$^{-1}$). The survival of bone marrow cells was assessed by the spleen colony assay. Results are means ± s.e. for six mice

200 mg kg$^{-1}$, caused no significant toxicity compared with untreated bone marrow cells (Figure 5). Cyclophosphamide proved to be more myelotoxic than either of the bioreductive agents causing a maximum cell kill of 1 log. The administration of AQ4N (100 mg kg$^{-1}$) or tirapazamine (25 mg kg$^{-1}$) 30 min prior to cyclophosphamide (100 mg kg$^{-1}$) resulted in some enhancement in bone marrow toxicity compared to cyclophosphamide (100 mg kg$^{-1}$) alone ($P$ = 0.001) (Figure 6). The effect of tirapazamine administered 24 h prior to cyclophosphamide also resulted in a significant ($P$ = 0.0005) enhancement of bone marrow toxicity. However, increasing the time interval for administration to 6 h between AQ4N and cyclophosphamide reduced bone marrow toxicity, compared to the effect observed at the 30 min time interval. In fact, the administration of AQ4N 6 h prior to cyclophosphamide did not significantly potentiate cyclophosphamide toxicity ($P$ = 0.895) (Figure 6).

### DISCUSSION

AQ4N has previously been shown to enhance the anti-tumour effect of both single and fractionated irradiation in the T50/80 tumour model (McKeown et al, 1995, 1996). The aim of this study was to determine if a similar enhancement could be achieved with the chemotherapy drug cyclophosphamide in the T50/80, SCCVII and RIF-1 tumour models systems. Tirapazamine was compared to AQ4N, since tirapazamine is the most successful bioreductive drug to enter clinical trials (Johnson et al, 1997).

In the T50/80 study a significant increase in the anti-tumour effect of AQ4N was observed when it was combined with a single dose of cyclophosphamide (100 mg kg$^{-1}$). The effect was found to occur at AQ4N doses as low as 50 mg kg$^{-1}$ ($P$ = 0.0001) and was independent of scheduling over a 6-h period (Figure 1). The anti-tumour effect of AQ4N combined with cyclophosphamide (100 mg kg$^{-1}$) was equivalent to that produced by a single 200 mg kg$^{-1}$ dose of cyclophosphamide alone (Figure 2), i.e. a 50% reduction in cyclophosphamide dose.

The additive effect of these two agents may result from a mechanism similar to that proposed for AQ4N and radiation. The cyclophosphamide will preferentially target cells that are oxygenated since it is a prodrug requiring cytochrome P450 mixed function oxidase activation. Upon activation, the resulting mustard is likely to alkylate DNA and other macromolecules in the close vicinity. In addition, cells closer to blood vessels will be preferentially exposed to higher doses and are more likely to be killed. AQ4N does not bind to DNA and will, therefore, diffuse through the tumour cell population until it enters a hypoxic cell, it will then become metabolized and the reduction product, AQ4, will bind to DNA (Patterson et al, 1993; McKeown et al, 1996; Smith et al, 1996). A possible explanation of the additive effect is that the drug combinations kill two distinct cell populations. A similar effect was observed with radiation where an increase in anti-tumour effect was reported when AQ4N was administered between 4 days before and 6 h after a single 12 Gy dose of radiation (McKeown et al, 1995).

When cyclophosphamide (100 mg kg$^{-1}$) was used in combination with tirapazamine it was necessary to reduce the tirapazamine dose to 50% of its maximum tolerated dose (i.e. 25 mg kg$^{-1}$) because of unacceptable weight loss observed with higher dose combinations. A significant increase in anti-tumour effect was observed with this combination (Figure 1), which was equivalent to a single 150 mg kg$^{-1}$ dose of cyclophosphamide alone (Figure 2). This result correlated well with three earlier studies combining tirapazamine and cyclophosphamide (Holden et al, 1992; Langmuir et al, 1994; Siemann et al, 1996). In all three studies, tirapazamine was found to enhance the anti-tumour effect of a single dose of cyclophosphamide and in two of the three

 © 2000 Cancer Research Campaign studies this was accompanied by an increase in systemic toxicity (Holden et al, 1992; Siemann 1996). Overall, in the T50/80 tumour model AQ4N was more effective than tirapazamine when combined with cyclophosphamide.

When drug combinations were examined in the SCCVII model, a clonogenic assay was used to measure anti-tumour efficacy. Again both bioreductive drugs enhanced the anti-tumour effect of cyclophosphamide with AQ4N showing a better anti-tumour effect than tirapazamine (Figure 3). Our results for tirapazamine, although not directly comparable, showed a similar level of enhancement to that reported in the RIF-1 tumour by Dorie and Brown (1997). A particularly impressive result was observed with AQ4N since for 6/9 tumours the number of clonogenic cells remaining after treatment was beyond the limits of detection of the assay, i.e. less than 1 in $5 \times 10^6$ cells were capable of forming colonies.

The results were equally impressive in the RIF-1 tumour model, again AQ4N increased the effect of cyclophosphamide alone by greater than 3 logs of cell kill. The time scheduling of AQ4N prior to cyclophosphamide did not influence the anti-tumour efficacy of the combination of the two drugs (Table 1). Tirapazamine, although reported to enhance the anti-tumour affect of cyclophosphamide in the RIF-1 model (Dorie and Brown, 1997), did not prove to be very successful in this particular study.

In order to assess the therapeutic potential of AQ4N it was necessary to evaluate normal tissue toxicity in a relevant systemic tissue. The bone marrow was selected since AQ4 is structurally similar to mitoxantrone; the major limiting toxicity of mitoxantrone is to the bone marrow. Cyclophosphamide is also myelotoxic and therefore it might be predicted that mitoxantrone, and possibly AQ4, would enhance the bone marrow toxicity of cyclophosphamide. In fact AQ4N significantly enhanced the anti-tumour effect of cyclophosphamide without increasing the toxicity of cyclophosphamide towards bone marrow cells when administered with a 6 h interval. In contrast, the administration of tirapazamine prior to cyclophosphamide resulted in a greater than additive toxicity at the 24 h interval. This result is in agreement with a previous report in which the effect of tirapazamine on cyclophosphamide toxicity was investigated (Siemann et al, 1996). When both drugs were given 30 min prior to cyclophosphamide there was some additional cell kill which may be relevant clinically. However, the enhanced toxicity had resolved by 6 h with AQ4N although it was still detectable with tirapazamine at the 24-h interval.

In conclusion AQ4N shows very considerable enhancement of the anti-tumour effect of cyclophosphamide. In comparison we found a more limited enhancement of the anti-tumour effect of cyclophosphamide with tirapazamine. However, evidence suggests that tirapazamine is more effective with cisplatin and it is currently showing efficacy in this combination in clinical trials (Johnson et al, 1997). Combination of AQ4N with cyclophosphamide shows little dependence on dose over 50–150 mg $kg^{-1}$ AQ4N and appears maximal for a schedule that includes dosing up to 24 h apart. If these results can be reproduced in the clinic AQ4N could provide appreciable enhancement of the anti-tumour efficacy of cyclophosphamide with no major enhancement of systemic toxicity.

### ACKNOWLEDGEMENTS

This research was funded by the Ulster Cancer Foundation. The authors would like to thank Miss Wendy Allen and Miss Lynne Murphy for their technical assistance in the experimental work carried out for this paper.

### REFERENCES

Adams GE, Stratford IJ, Edwards HS, Bremner JCM and Cole S (1992) Bioreductive drugs as post-irradiation sensitizers: comparison of dual function agents SR4233 and the mitomycin C analogue EO9. *Int J Radiat Oncol Biol Phys* **22**: 717–720

Brown JM and Lemmon MJ (1990) Potentiation by the hypoxic cytotoxin SR4233 of cell killing produced by fractionated irradiation of mouse tumours. *Cancer Res* **50**: 7745–7749

Dorie MJ and Brown JM (1997) Modification of the anti-tumour activity of chemotherapy drugs by the hypoxic cytotoxic agent tirapazamine. *Cancer Chemother Pharmacol* **39**: 361–266.

Holden SA, Teicher BA, Ara G, Herman TS and Coleman CN (1992) Enhancement of alkylating agent activity by SR4233 in the FSaIIC murine fibrosarcoma. *J Natl Cancer Inst* **84**: 187–193

Johnson CA, Kilpatrick D, von Roemeling R, Langer C, Graham MA, Greenslade D, Kennedy G, Keenan E and O'Dwyer PJ (1997) Phase I trial of tirapazamine in combination with cisplatin in a single dose every three weeks in patients with solid tumours. *Br J Cancer* **73**: 1480–1485

Kennedy KA (1987) Hypoxic cells as specific drug targets for chemotherapy. *Anticancer Drug Des* **2**: 181–194

Langmuir VK, Rooker JA, Osen M, Mendonca HL and Laderoute KR (1994) Synergistic interaction between tirapazamine and cyclophosphamide in human breast cancer xenografts. *Cancer Res* **54**: 2845–2847

McAleer JJA, McKeown SR, MacManus MP, Lappin TRJ and Bridges JM (1992) Hypobaric hypoxia: a method for testing bioreductive drugs in vivo. *Int J Radiat Oncol Biol Phys* **23**: 551–555

McKeown SR, Hejmadi MV, McIntyre IA, McAleer JJA and Patterson LH (1995) AQ4N: an alkylaminoanthraquinone N-oxide showing bioreductive potential and positive interaction with radiation in vivo. *Br J Cancer* **71**: 76–81

McKeown SR, Friery OP, McIntyre IA, Hejmadi MV, Patterson LH and Hirst DG (1996) Evidence for a therapeutic gain when AQ4N or tirapazamine is combined with radiation. *Br J Cancer* **74**: S39–S42

Moore JV (1988) The dynamics of tumour cords in an irradiated mouse mammary carcinoma with a large hypoxic cell component. *Jpn J Cancer Res (Gann)* **79**: 236–243

Patterson LH (1993) Rationale for the use of aliphatic N-oxides of cytotoxic anthraquinones as prodrug DNA binding agents: a new class of bioreductive agent. *Cancer Metast Rev* **12**: 119–134

Siemann DW (1994) In vitro cytotoxicity and chemosensitizing activity of the dual function nitroimidazole RB6145. *Int J Radiat Oncol Biol Phys* **29**: 301–306

Siemann DW (1996) The in situ tumour response to combinations of cyclophosphamide and tirapazamine. *Br J Cancer* **74**: S65–S69

Smith PJ, Blunt NJ, Desnoyers R, Giles Y and Patterson LH (1996) DNA topoisomerase II dependent cytotoxicity of alkylaminoanthraquinones and their N-oxides. *Cancer Chemother Pharmacol* **39**: 455–461

Till JE and McCulloch EA (1961) A direct measurement of the radiation sensitivity of normal mouse bone marrow cells. *Radiat Res* **14**: 213–222

Workman P and Stratford IJ (1993) The experimental development of bioreductive drugs and their role in cancer therapy. *Cancer Metast Rev* **12**: 73–82

© 2000 Cancer Research Campaign
